# Integrative genomic analysis reveals widespread enhancer regulation by p53 in response to DNA damage

**DOI:** 10.1093/nar/gkv284

**Published:** 2015-04-16

**Authors:** Scott T. Younger, Daniela Kenzelmann-Broz, Heiyoun Jung, Laura D. Attardi, John L. Rinn

**Affiliations:** 1 Department of Stem Cell and Regenerative Biology, Harvard University, Cambridge, MA 02138, USA; 2 Broad Institute of MIT and Harvard, Cambridge, MA 02142, USA; 3 Division of Radiation and Cancer Biology, Department of Radiation Oncology, Stanford University School of Medicine, Stanford, CA, USA; 4 Department of Pathology, Beth Israel Deaconess Medical Center, Boston, MA 02215, USA

## Abstract

The tumor suppressor p53 has been studied extensively as a direct transcriptional activator of protein-coding genes. Recent studies, however, have shed light on novel regulatory functions of p53 within noncoding regions of the genome. Here, we use a systematic approach that integrates transcriptome-wide expression analysis, genome-wide p53 binding profiles and chromatin state maps to characterize the global regulatory roles of p53 in response to DNA damage. Notably, our approach identified conserved features of the p53 network in both human and mouse primary fibroblast models. In addition to known p53 targets, we identify many previously unappreciated mRNAs and long noncoding RNAs that are regulated by p53. Moreover, we find that p53 binding occurs predominantly within enhancers in both human and mouse model systems. The ability to modulate enhancer activity offers an additional layer of complexity to the p53 network and greatly expands the diversity of genomic elements directly regulated by p53.

## INTRODUCTION

The tumor suppressor p53 is an essential cellular stress sensor and a central line of defense against genomic instability (1–6). In response to DNA damage p53 is stabilized and acts as a transcription factor that directly regulates several hundred genes and indirectly regulates thousands more (2). Traditionally, p53 has been thought to act through direct transcriptional regulation of protein-coding genes. However, recent reports have demonstrated functional roles for p53 that extend beyond the coding genome.

One recently appreciated function of p53 within the noncoding genome is the ability to regulate expression of long noncoding RNAs (lncRNAs). Several lncRNAs have been characterized as functionally important targets of p53 in both mouse and human models (7,8). Among these RNAs is lincRNA-p21, a lncRNA that has revealed a diversity of functional roles ranging from regulation of apoptosis to translational suppression of complementary mRNAs (9,10). Another p53-regulated lncRNA, Pint, has been shown to bind to Polycomb repressive complex 2 (PRC2) and direct epigenetic silencing of genes involved with cellular growth and proliferation in response to DNA damage (11). The well-known oncogenic noncoding RNA PVT1 has also been identified as a direct target of p53 in human cells, although the exact functional role of PVT1 in the DNA damage response remains unclear (12). The study of p53-regulated lncRNAs is still in its infancy and these examples likely represent a small subset of a larger class of lncRNAs that has yet to be fully characterized.

Another recently uncovered role of p53 within the noncoding genome is the recognition of regulatory enhancer elements. An analysis of p53 binding events in human fibroblasts identified seven sites that occur within enhancer-like regions (13). These p53-bound enhancers regulate the expression of multiple genes over long distances via chromosome looping. A detailed study of a single p53-bound enhancer in *Drosophila* yielded similar results (14). These common observations in distant organisms support the hypothesis that enhancer regulation may be a general function of p53. However, the aforementioned studies were focused on isolated genomic loci and a comprehensive analysis of enhancer recognition by p53 has not been performed to date.

Here, we extend beyond previous genome-scale studies on p53 regulation using a multifaceted and systematic approach that integrates transcriptome-wide differential expression analysis, genome-wide p53 binding profiles, chromatin state maps and additional genomic features to interrogate the global regulatory functions of p53 in response to DNA damage. Furthermore, we have performed these analyses in orthologous human and mouse fibroblast models to better understand the conserved and divergent properties of the p53 regulatory network across mammals. Collectively, these data provide an unprecedented and comprehensive overview of the p53 response to DNA damage in normal, untransformed primary cells from both human and mouse.

In addition to p53-regulated genes identified in prior studies, our approach has uncovered many previously unappreciated transcriptional targets of p53, including both protein-coding genes and lncRNAs. The sequences of p53-regulated lncRNAs are distinguished from the global transcriptome in both human and mouse by their transposable element (TE) composition, suggesting that specific TE sequences may influence the functional roles of these noncoding RNAs in the DNA damage response. Interestingly, the majority of p53 binding sites we detected fall within intergenic regions not associated with direct transcriptional regulation. The chromatin environment surrounding these binding sites revealed that they are significantly enriched within regulatory enhancer elements. Moreover, p53 binding sites occur much more frequently within annotated enhancers than within regions associated with transcription start sites. Enhancers bound by p53 display a distinct transcription factor co-occupancy landscape that indicates p53 likely functions as a dominant regulator of enhancer activity. Altogether, these multidimensional analyses emphasize the importance of p53 beyond the direct transcriptional regulation of protein-coding genes and reveal that regulation of enhancer elements is a predominant feature of the p53 response to DNA damage.

## MATERIALS AND METHODS

### Acquisition of publically available data sets

Repeat element sequences for both the human and mouse genomes were obtained through the RepeatMasker tracks from the UCSC Genome Browser. Histone ChIP-Seq data sets used to generate chromatin state maps were obtained from the Roadmap Epigenomics Project and the UCSC Genome Browser. Human transcription factor binding sites were obtained through the transcription factor ChIP-Seq Clusters Version 3 track from the UCSC Genome Browser. Mouse histone ChIP regions and transcription factor binding sites were obtained through the Ludwig Institute for Cancer Research (LICR) tracks from the UCSC Genome Browser.

### Cell culture

Human fetal fibroblast lines GM00011 and GM06170 (Coriell Cell Repositories) were cultured in Dulbecco's modified Eagle's medium (DMEM) supplemented with 15% Fetal Bovine Serum (Life Technologies). MEFs were generated from E13.5 embryos and cultured in DMEM supplemented with 10% Fetal Bovine Serum. Human and mouse fibroblasts were treated with 0.2 ug/ml doxorubicin for 12 h and 6 h, respectively, to induce DNA damage.

### RNA isolation and quantitative PCR

RNA from treated fibroblasts was isolated using TRIzol (Life Technologies) as per the manufacturer's instructions. For each sample, 2 μg of RNA was reverse transcribed using SuperScript III Reverse Transcriptase (Life Technologies). RNA was treated with DNase I (Worthington) prior to reverse transcription. qPCR was performed on an ABI7900HT real-time PCR (Applied Biosystems) using FastStart Universal SYBR Green Master—Rox (Roche). Primers for TBP mRNA were supplied by Applied Biosystems. All additional primers were designed using Primer3. Only those primer sets that showed linear amplification over several orders of magnitude were used for quantification. Primers and PCR conditions are listed in Supplementary Table S2.

### Chromatin Immunoprecipitation

Human fibroblasts were crosslinked with 1% formaldehyde in PBS (10 ml) for 10 min. The crosslinking reaction was quenched with glycine (0.125M final concentration) for 5 min. Cells were collected and nuclei were isolated by two successive washes with 5 ml cold hypotonic lysis buffer (10 mM Tris pH 7.4, 10 mM NaCl, 3 mM MgCl_2_, 0.5% Nonidet P40). Pelleted nuclei were lysed in 1 ml cold nuclear lysis buffer (10 mM Tris pH 7.5, 1% Nonidet P40, 0.5% sodium deoxycholate, 0.1% SDS, 1X Protease Inhibitor Cocktail) and flash frozen in liquid nitrogen. DNA was sonicated on ice to an average fragment size of ∼500 bp followed by centrifugation to remove insoluble cell debris. Aliquots of lysate (100 ul) were diluted to 1 ml in IP buffer (0.01% SDS, 1.1% Triton X-100, 1.2 mM EDTA, 16.7 mM Tris pH 8.0, 167 mM NaCl, 1× Protease Inhibitor Cocktail). Individual aliquots were precleared for 1 hr with Proteain A/G Dynabeads (Life Technologies). Antibodies were added to each aliquot (4 ug/aliquot) and incubated at 4°C overnight. Protein A/G Dynabeads (50 ul) were added to precipitate the antibody complexes and the beads were successively washed with 1 ml of the following wash buffers: low-salt (0.1%SDS, 1% Triton X-100, 2 mM EDTA, 20 mM Tris pH 8.0, 150 mM NaCl), high-salt (0.1%SDS, 1% Triton X-100, 2 mM EDTA, 20 mM Tris pH 8.0, 500 mM NaCl), LiCL (0.25 M LiCl, 1% Nonidet P40, 1% sodium deoxycholate, 1 mM EDTA, 10 mM Tris pH 8.0). Beads were then washed twice with 1 ml TE pH 8.0. Complexes were eluted from beads with two successive 250 ul incubations in elution buffer (1% SDS, 0.1M NaHCO_3_). NaCl was added (final concentration 200 mM) and the samples heated to 65°C overnight to reverse crosslinks. The samples were then treated with RNase A (1 ul) at 37°C for 30 min, followed by the addition of Tris pH 7.0 (20 ul), 0.5 M EDTA (10 ul), and proteinase K (20 ug) and incubation at 42°C for 45 min. DNA was isolated by phenol-chloroform extraction and isopropanol precipitation. DNA pellets were resuspended in 50 ul water. For ChIP-qPCR 2 ul of sample was used per reaction and enrichment was calculated by comparison with 1% of the corresponding input sample. α-p53 (2524S), α-Phospho-p53-Ser15 (9284S) and normal IgG (2729S) antibodies were supplied by Cell Signaling. α-H3K4me1 (07–436) and α-H3K27ac (07–442) antibodies were supplied by Millipore. Primers for lincRNA promoters and enhancers were designed using Primer3. Only those primer sets that showed linear amplification over several orders of magnitude were used for quantification. Primers and PCR conditions are listed in Supplementary Table S2.

### RNA isolation and sequencing library construction

RNA from treated fibroblasts was isolated using TRIzol (Life Technologies) as per the manufacturer's instructions. RNA was collected from two biological replicates for human fibroblasts and three biological replicates for mouse fibroblasts. RNA sequencing libraries were prepared with the TruSeq RNA Sample Preparation Kit (Illumina) as per the manufacturer's instructions using 500 ng of input RNA for each library. Prior to sequencing, the quality and concentration of each library was assessed using the Bioanalyzer (Agilent).

### RNA sequencing and transcript alignment

RNA-Seq libraries were sequenced on a HiSeq 2000 (Illumina). For characterization of protein-coding genes, sequencing reads were mapped to UCSC known genes for human (hg19) or mouse (mm9) using TopHat2 with default options (15). For lincRNA characterization, sequencing reads were mapped to our previously described catalogs (generated from compendiums of human or mouse cell lines) using TopHat2 with default options (16).

### Gene expression analysis

Differential expression of protein-coding genes and lincRNAs in response to treatment with doxorubicin was assessed using Cuffdiff2 with default options. The subset of differentially expressed protein-coding genes classified as significant by Cuffdiff2 was further restricted to the top 75% of expressed genes (based on FPKM values). The subset of differentially expressed lincRNAs classified as significant by Cuffdiff2 was further restricted to those with FPKM values greater than 1 in at least one sample.

### ChIP-Seq and ChIP-Seq analysis

ChIP experiments and ChIP-Seq library preparation were performed as previously described (17). ChIP experiments were carried out using a monoclonal antibody against p53 (DO-1, Santa Cruz) in human fibroblasts and a polyclonal antibody against p53 (CM5, Vector Laboratories) in MEFs. ChIP-Seq libraries were sequenced on a Genome Analyzer II (Illumina). ChIP-Seq reads were aligned to the human (hg19) or mouse (mm9) genomes using Bowtie2 (18). Aligned reads were analyzed using Scripture (Broad) to generate a catalog of potential ChIP peaks (19). To identify high confidence ChIP peaks, differential read coverage between ChIP samples and their respective input samples was assessed with Cuffdiff2 using the Scripture output as a reference annotation. Peak intensities are represented using FPKM, which indicates ‘Fragments Per Kilobase Of ChIP-Peak Per Million Fragments Mapped’. High confidence ChIP peaks were further filtered to the top 75% of read-covered peaks (based on FPKM values in the ChIP samples). MEME-ChIP was used to analyze the presence and distribution of sequence motifs within ChIP peaks (20).

### LincRNA: guilt by association and repeat sequence analysis

For guilt by association a pre-ranked list for each candidate RNA was generated consisting of protein-coding genes with similar expression profiles across a compendium of cell/tissue types, with each gene ranked by its Jensen Shannon distance from the RNA of interest (16). The resulting ranked list was subjected to Gene Set Enrichment Analysis (Broad) using the Reactome gene sets from the Molecular Signatures Database (21). For sequence motif identification, the exon sequences of selected lincRNAs were analyzed with MEME using the default settings (22). Enrichment of selected repeat elements was calculated by dividing the normalized number of repeat element occurrences within the selected subset of RNAs by the normalized number of occurrences within the transcriptome. For normalization the number of occurrences within a data set (select RNAs or transcriptome) was divided by the following ratio: nucleotide coverage of the data set/nucleotide coverage of the selected repeat element. To calculate statistical significance of enrichment the labels of all RNAs within the transcriptome were randomly shuffled and the analysis was repeated 100 times. The resulting *p*-values were corrected for multiple hypothesis testing using the Bonferroni method.

### Chromatin state map generation

To generate a general chromatin state map for human fibroblasts the state maps for several cell types were intersected, resulting in a single map consisting of states that were common to all cell types selected. The cell types selected for this intersection were two unique human foreskin fibroblast samples, two unique human foreskin keratinocyte samples and normal human epidermal keratinocytes (NHEKs). Chromatin state maps were generated from existing histone ChIP-Seq data for each individual cell type using ChromHMM (23). The specific histone marks used to generate state maps for foreskin fibroblasts and foreskin keratinocytes were H3K27ac, H3K27me3, H3K36me3, H3K4me1, H3K4me3 and H3K9me3. The state map for NHEKs also included H3K9ac. Histone ChIP-Seq data sets for foreskin fibroblasts and foreskin keratinocytes were acquired from the Roadmap Epigenomics Project. Histone ChIP-Seq data for NHEKs were acquired from the UCSC Genome Browser.

### Chromatin state enrichment analyses

Enrichment of chromatin states was calculated by dividing the normalized number of ChIP peaks overlapping a given state by the number of peaks in the genome. For normalization the number of ChIP peak overlaps within a data set (chromatin state or whole genome) was divided by the following ratio: nucleotide coverage of the data set/nucleotide coverage of ChIP peaks. To calculate statistical significance of enrichment the labels of all chromatin states were randomly shuffled and the analysis was repeated 100 times. The resulting *p*-values were corrected for multiple hypothesis testing using the Bonferroni method. The same analysis was applied to mouse data with the exception that modified histone ChIP-Seq peaks were used in place of chromatin state maps.

### Transcription factor binding site enrichment analyses

For analysis of transcription factor binding sites within p53-bound enhancers the annotated binding sites for each transcription factor were intersected with the previously generated enhancer state map, resulting in a list of bound enhancers for each respective factor. Enrichment of transcription factor binding site occurrence was calculated by dividing the normalized number of p53-bound enhancers in common with a given transcription factor by the normalized number of p53-bound enhancers within the genome. For normalization the number of enhancers within a data set (common enhancers or all enhancers) was divided by the following ratio: nucleotide coverage of the data set/nucleotide coverage of all p53-bound enhancers. To calculate statistical significance of enrichment the labels of all transcription factor-bound enhancers were randomly shuffled and the analysis was repeated 100 times. The resulting *p*-values were corrected for multiple hypothesis testing using the Bonferroni method. Significance analysis of global transcription factor binding site co-occurrence within enhancers was calculated using the hypergeometric distribution given the number of enhancers overlapped by each factor under comparison and the total number of enhancers in the genome. Resulting *p*-values were corrected for multiple hypothesis testing using the Bonferroni method.

## RESULTS

### Identification of protein-coding genes that are directly regulated by p53 in response to DNA damage in primary human fibroblasts

**Figure 1.**
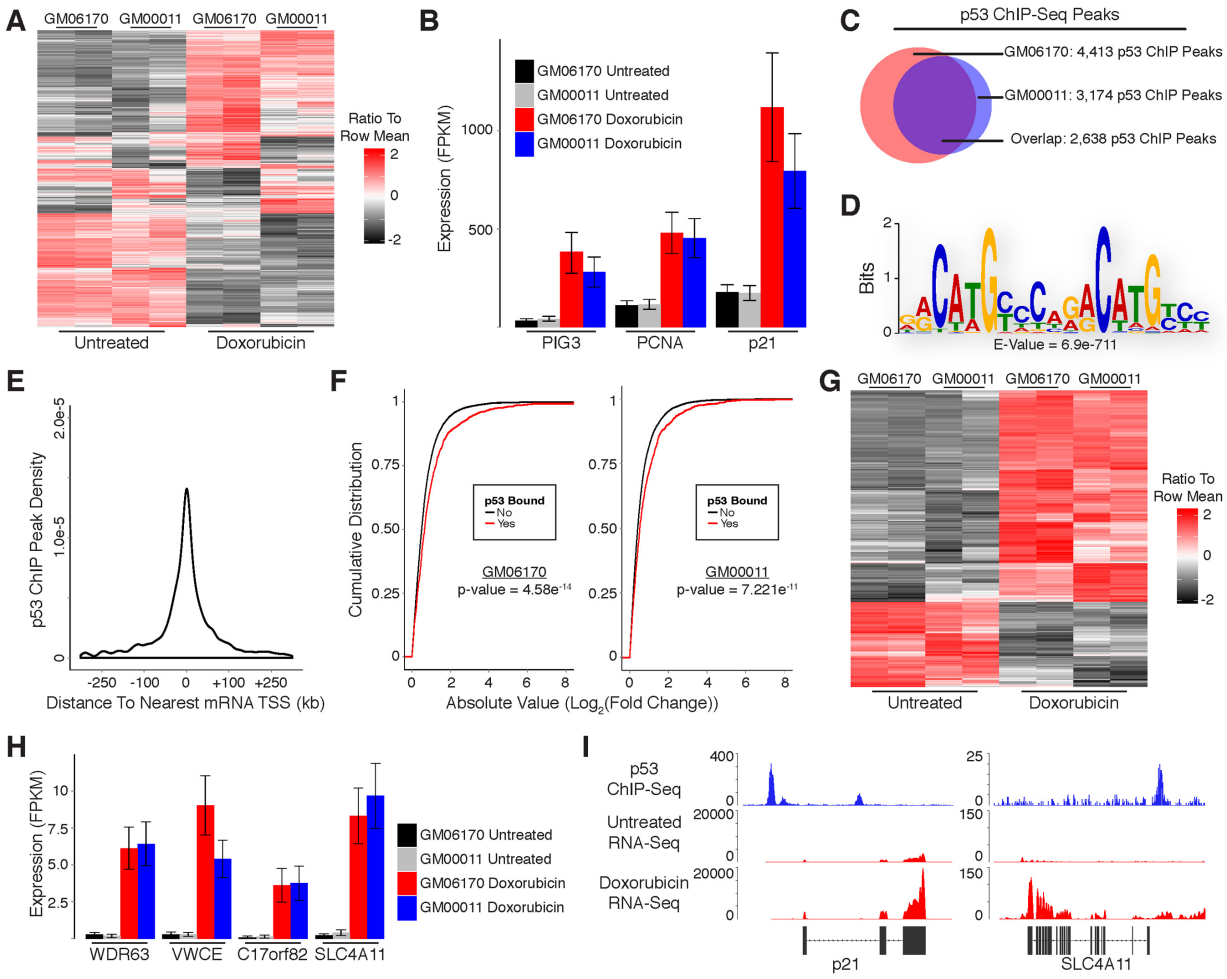
Identification of protein-coding genes that are directly regulated by p53 in response to DNA damage in primary human fibroblasts. (**A**) Heatmap showing differential expression of protein-coding mRNAs in response to DNA damage. (**B**) RNA-Seq analysis of canonical p53 target genes in response to DNA damage. (**C**) Overlap of p53 ChIP-Seq peaks from GM06170 and GM00011 fibroblasts. (**D**) Sequence motif analysis of p53 ChIP peaks. (**E**) Distribution of p53 ChIP peaks relative to transcription start sites of protein-coding genes. (**F**) Cumulative distribution of expression changes for all p53 bound and unbound mRNAs in response to DNA damage. (**G**) Heatmap showing differential expression of direct p53 target mRNAs in response to DNA damage. (**H**) RNA-Seq analysis of previously uncharacterized p53 target genes in response to DNA damage. (**I**) RNA-Seq and p53 ChIP-Seq at selected loci. *P*-values (**1F**) were calculated using the Kolmogorov-Smirnov test.

As a model system to study transcriptional regulation by p53 in response to DNA damage we treated cultured primary human fibroblasts with the DNA double-strand break inducing agent doxorubicin. To recapitulate a normal p53-dependent response to DNA damage we selected two untransformed human fetal fibroblast lines, GM00011 and GM06170. The use of multiple cell lines also facilitates the discrimination of general p53 properties from cell line-specific observations. We isolated RNA from fibroblasts cultured in the presence or absence of doxorubicin for 12 h and performed paired-end RNA-Sequencing (RNA-Seq) with an average sequencing depth of 31.6 million mapped fragments per sample. We then evaluated the differential expression of protein-coding genes in response to DNA damage using the UCSC known genes annotation as a reference transcriptome. Treatment with doxorubicin resulted in up-regulation of 1365 mRNAs and down-regulation of 1598 mRNAs common to both cell lines (Figure 1A; Supplementary Table S1). Included in the list of up-regulated mRNAs were well-established p53 target genes such as p21, PCNA and PIG3 (Figure 1B).

While the DNA damage response results in differential expression of many genes, only a subset of these are directly regulated by p53. To further distinguish direct p53 target genes from those that are indirectly affected by DNA damage we performed chromatin immunoprecipitation (ChIP) in doxorubicin-treated fibroblasts using antibodies against p53. Isolated DNA was subjected to single-end sequencing with an average depth of 21.9 million uniquely mapped fragments per sample. ChIP-Sequencing (ChIP-Seq) identified 4413 and 3174 p53 binding sites in GM06170 and GM00011 cells, respectively. Comparison of p53 binding sites between fibroblast lines revealed 2638 sites common to both lines (Figure 1C). Motif analysis of the common p53 ChIP peaks using Multiple Em for Motif Elicitation (MEME), specifically MEME-ChIP, revealed enrichment of the p53 consensus binding motif, confirming the specificity of the ChIP experiment for p53 binding sites (Figure 1D) (20). In addition, we performed a highly stringent search for p53 binding motifs (specifically [A/G]C[A/T][A/T]G[C/T][C/T][2–4 Spacer Nucleotides][G/A][G/A]C[A/T][A/T]G[T/C]) within the p53 ChIP peaks common to both fibroblast lines and identified clear motifs in 1074 of the 2638 peaks. The remaining peaks may have p53 motifs that deviate slightly from our criteria. Alternatively, these peaks could arise indirectly from distal regulatory elements that contain p53 motifs interacting with their genomic targets (which may lack canonical p53 motifs).

To further validate our ChIP-Seq results we compared the p53 binding sites we identified to those described in previous studies that performed p53 ChIP-Seq (24–26). We observed appreciable, albeit incomplete, overlap in p53 binding sites between our data and the majority of data sets we analyzed (Supplementary Figure S1A–C). Binding sites characterized in cells treated with doxorubicin (lymphoblastoid or U2OS) displayed the most overlap with our data (Supplementary Figure S1D). Altogether, 93% of the p53 binding sites identified in our study were present in at least one other data set. In contrast to the untransformed fibroblasts from our study, these alternative studies used cancer cell line models (MCF7, lymphoblastoid, or U2OS cells). Moreover, they utilized a variety of methods for activating p53 as well as different antibodies in their ChIP protocols. These observations indicate that our ChIP-Seq results are not biased by the type of cells or antibodies used in our experiments and are representative of a general response to DNA damage by p53.

Unlike traditional transcription factors that bind within a few hundred base pairs of transcription start sites (TSSs), p53 has been shown in some cases to bind many kilobases (kb) up- or downstream of its target genes. We empirically evaluated the relationship between p53 ChIP peaks and protein-coding gene TSSs, finding that most p53 binding sites occur within 30 kb of an annotated TSS (Figure 1E). Moreover, genes with p53 binding sites within 30 kb of their TSSs had significantly higher levels of induction/repression in response to DNA damage as compared to genes without binding sites, further supporting that they are directly regulated by p53 (Figure 1F). Based on these results we intersected p53 ChIP peaks with genomic regions spanning from 30 kb upstream to 30 kb downstream of TSSs for genes that were differentially expressed in response to DNA damage, identifying a small subset of 262 up-regulated and 106 down-regulated genes that are direct targets of p53 (Figure 1G; Supplementary Table S1; Supplementary File 1). In contrast to the noticeable level of variation in global gene expression between GM06170 and GM00011 cells (Figure 1A), direct p53 target genes display consistent expression across cell lines (Figure 1G). The association of p53 with most of these genes has been previously unappreciated, possibly because many have relatively low expression compared to canonical p53 target genes (Figure 1H, Supplementary File 1). RNA-Seq and p53 ChIP-Seq at the p21 locus (a well-known p53 target gene) and SLC4A11 locus (an anti-apoptotic gene not previously associated with p53) are shown in Figure 1I (27).

### Identification of protein-coding genes that are directly regulated by p53 in response to DNA damage in primary mouse embryonic fibroblasts

The p53 protein, as well as its transcriptional regulation of protein-coding gene targets, is highly conserved throughout evolution (28). We reasoned that a systematic evaluation of the DNA damage response in mouse fibroblasts might shed light on the conserved functions of p53 within the noncoding genome. We generated primary mouse embryonic fibroblasts (MEFs) and treated them with doxorubicin for 6 h to induce DNA damage. RNA was isolated from MEFs and subjected to paired-end RNA-Seq with an average sequencing depth of 29 million mapped fragments per sample. We then evaluated the differential expression of protein-coding genes in response to DNA damage using the UCSC known genes annotation as a reference transcriptome.

**Figure 2.**
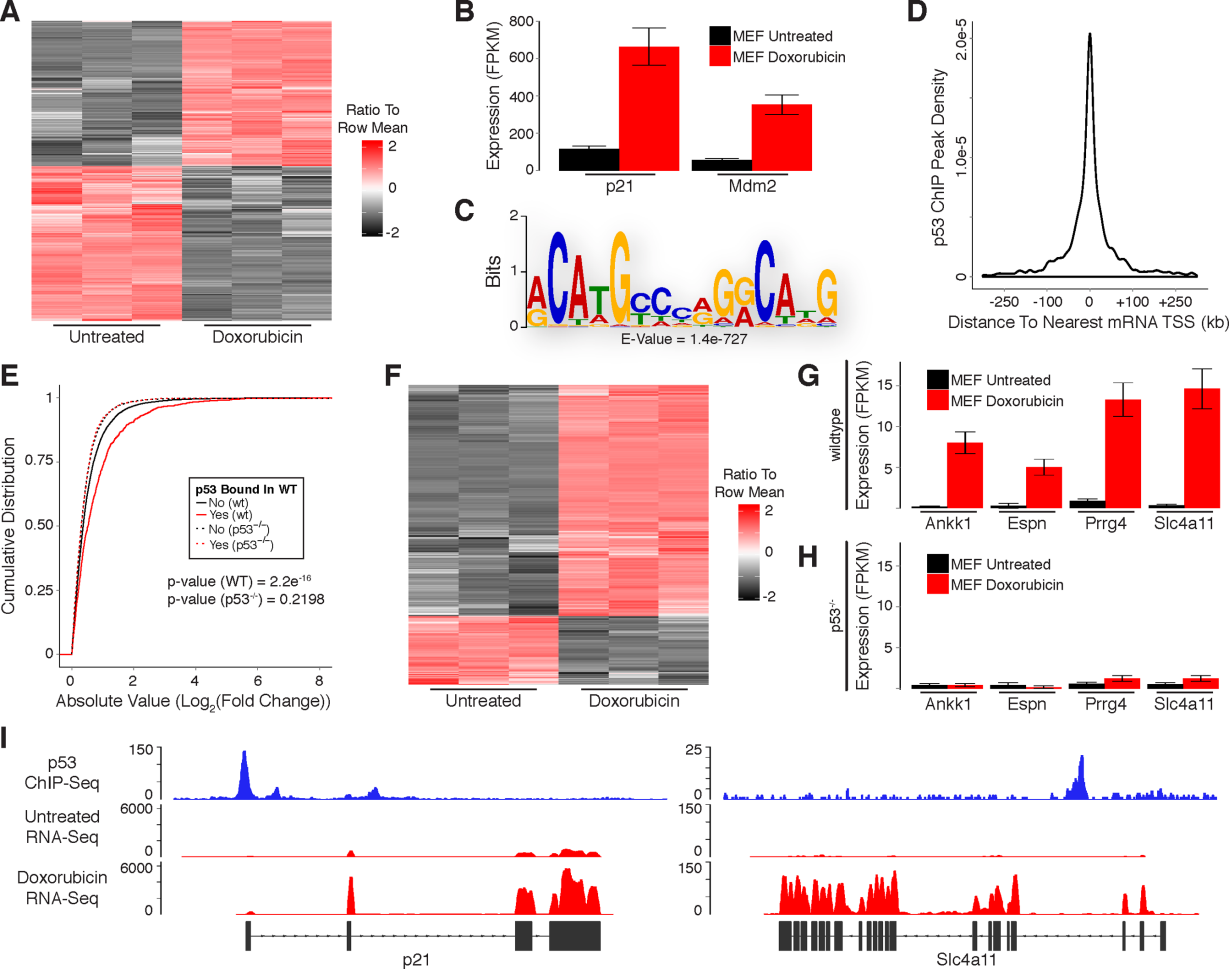
Identification of protein-coding genes that are directly regulated by p53 in response to DNA damage in primary mouse embryonic fibroblasts. (**A**) Heatmap showing differential expression of protein-coding mRNAs in response to DNA damage. (**B**) RNA-Seq analysis of canonical p53 target genes in response to DNA damage. (**C**) Sequence motif analysis of p53 ChIP peaks. (**D**) Distribution of p53 ChIP peaks relative to transcription start sites of protein-coding genes. (**E**) Cumulative distribution of expression changes for all p53 bound and unbound mRNAs in response to DNA damage in wild-type and p53^−/−^ primary MEFs. (**F**) Heatmap showing differential expression of direct p53 target mRNAs in response to DNA damage. (**G** and **H**) RNA-Seq analysis of previously uncharacterized p53 target genes in response to DNA damage in (**G**) wild-type and (**H**) p53^−/−^ primary MEFs. (**I**) RNA-Seq and p53 ChIP-Seq at selected loci. *P*-values (**2E**) were calculated using the Kolmogorov-Smirnov test.

Doxorubicin treatment resulted in up-regulation of 3124 mRNAs and down-regulation of 3334 mRNAs in MEFs (Figure 2A; Supplementary Table S1). Included in the list of up-regulated mRNAs were known p53 target genes such as p21 and Mdm2 (Figure 2B). As in human cells, only a subset of the mouse genes that are differentially expressed in response to DNA damage represent direct targets of p53. To further distinguish direct target genes we used a previously published ChIP-Seq data set that we generated using antibodies against p53 in doxorubicin-treated MEFs and identified 3100 binding sites (17). MEME-ChIP analysis of p53 ChIP peaks revealed enrichment of the p53 consensus binding motif, confirming the specificity of the ChIP experiment for p53 binding sites (Figure 2C). A highly stringent search for p53 binding motifs (using the previously described criteria) within the p53 ChIP peaks in MEFs also identified clear motifs in 900 of the 3100 peaks.

Most of the identified p53 binding sites occur within 20 kb of an annotated protein-coding gene TSS (Figure 2D). Furthermore, genes with p53 binding sites within these regions had significantly higher levels of induction/repression in response to DNA damage as compared to genes without binding sites (Figure 2E). Importantly, genes bound by p53 (in wild-type MEFs) were not induced/repressed in p53^−/−^ MEFs, demonstrating that they are directly regulated by p53 (Figure 2E). Upon the intersection of p53 ChIP peaks with genomic regions spanning from 20 kb upstream to 20 kb downstream of TSSs for genes that were differentially expressed in response to DNA damage we identified a subset of 585 up-regulated and 175 down-regulated genes that are direct targets of p53 (Figure 2F; Supplementary Table S1; Supplementary File 1). Our analysis uncovered many genes that were previously uncharacterized as direct targets of p53, including orthologs of several genes detected in our analysis of human fibroblasts (Figure 2G). Importantly, these genes were not induced in p53^−/−^ MEFs in response to DNA damage, confirming the ability of our approach to properly identify p53-regulated transcripts (Figure 2H). RNA-Seq and p53 ChIP-Seq at the p21 locus (a well-known p53 target gene) and Slc4a11 locus (an ortholog of the previously described human SLC4A11) are shown in Figure 2I.

### p53 regulates expression of many lincRNAs in response to DNA damage in primary human fibroblasts

**Figure 3.**
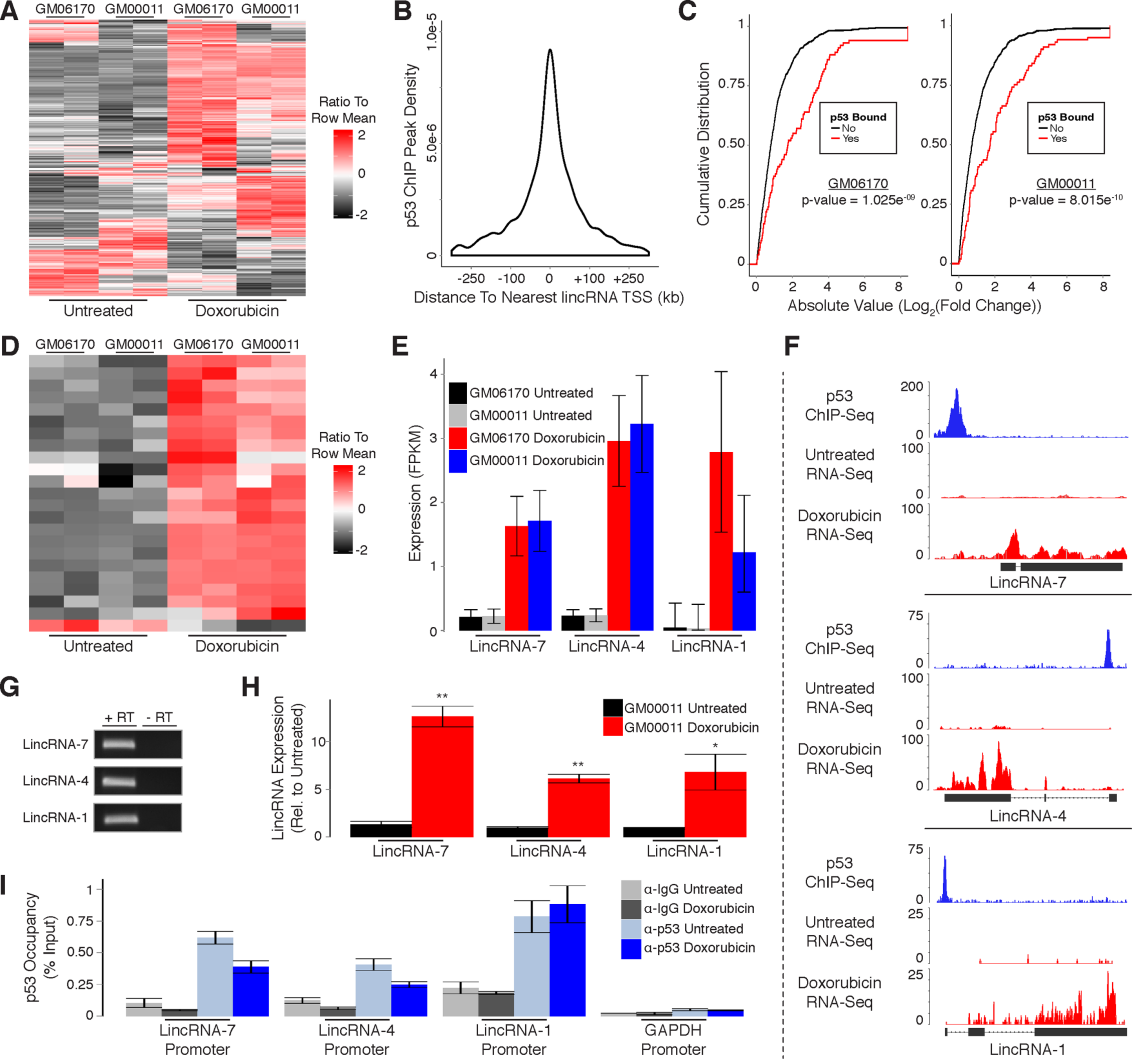
p53 regulates expression of many lincRNAs in response to DNA damage in primary human fibroblasts. (**A**) Heatmap showing differential expression of lincRNAs in response to DNA damage. (**B**) Distribution of p53 ChIP peaks relative to transcription start sites of lincRNAs. (**C**) Cumulative distribution of expression changes for all p53 bound and unbound lincRNAs in response to DNA damage. (**D**) Heatmap showing differential expression of direct p53 target lincRNAs in response to DNA damage. (**E**) RNA-Seq analysis of p53 target lincRNAs in response to DNA damage. (**F**) RNA-Seq and p53 ChIP-Seq at selected lincRNA loci. (**G**) RT-PCR detection of p53 target lincRNAs in GM00011 cells. (**H**) RT-qPCR showing induction of p53 target lincRNAs in response to DNA damage. (**I**) ChIP-qPCR analysis of p53 occupancy at lincRNA promoters in response to DNA damage. *P*-values (**3C**) were calculated using the Kolmogorov-Smirnov test. Error bars (**3H** and **3I**) indicate SEM (*n* = 3). *P*-values (**3H** and **3I**) were calculated using the two-tailed unpaired Student's *t*-test with equal variances. *P < 0.05, **P < 0.01.

Having characterized the protein-coding gene targets of p53 in response to DNA damage, we began evaluating p53's functional roles within the noncoding genome. We first examined the effects of DNA damage on noncoding RNA expression, specifically long intergenic noncoding RNAs (lincRNAs). To characterize the differential expression of lincRNAs in response to DNA damage we aligned the RNA-Seq data generated from human fibroblasts to our previously reported comprehensive lincRNA reference catalog (16). Doxorubicin treatment resulted in up-regulation of 101 lincRNAs and down-regulation of 32 lincRNAs common to both cell lines (Figure 3A; Supplementary Table S1).

We used p53 ChIP-Seq to distinguish direct target lincRNAs from those that are indirectly affected by DNA damage. In contrast to protein-coding genes, most p53 binding sites occur within 45 kb of the nearest lincRNA TSS (Figure 3B). However, to maintain consistency with our previous analysis of protein-coding gene regulation in human fibroblasts we performed all subsequent analyses with genomic regions spanning from 30 kb upstream to 30 kb downstream of TSSs for annotated lincRNAs. LincRNAs with p53 binding sites within these regions had significantly higher levels of induction/repression in response to DNA damage as compared to lincRNAs without binding sites, supporting that they are directly regulated by p53 (Figure 3C). Similar to the ratio of p53 bound to unbound protein-coding genes, we identified a subset of 22 up-regulated and only 1 down-regulated lincRNA that are direct targets of p53 (Figure 3D; Supplementary Table S1; Supplementary File 1). A small fraction of the p53 binding sites associated with lincRNA regulation (3/23) were also associated with the regulation of transcripts characterized in our analysis of protein-coding genes in human fibroblasts (Supplementary File 1).

Next, we randomly selected three of the p53-regulated lincRNAs we identified for experimental validation. While these lincRNAs had relatively low expression levels compared to protein-coding genes, they were reproducibly induced in both human fibroblast lines in response to DNA damage (Figure 3E; Supplementary File 1). Moreover, our RNA-Seq results closely resemble the transcript structures of the lincRNA annotations within our reference catalog (Figure 3F). To experimentally confirm the existence of these lincRNAs we extracted RNA from doxorubicin treated GM00011 fibroblasts and performed RT-PCR (Figure 3G). We then performed quantitative RT-PCR (RT-qPCR) and observed significant induction of all three lincRNAs in response to doxorubicin treatment in GM00011 fibroblasts (Figure 3H). We also quantified p53 occupancy at the promoters of all three lincRNAs using ChIP-qPCR in GM00011 fibroblasts cultured in the presence or absence of doxorubicin. Interestingly, p53 was present at each lincRNA promoter prior to doxorubicin treatment (Figure 3I). These results confirm that the lincRNAs we selected for experimental validation are regulated by p53 and suggest a model whereby p53 is actively poised at lincRNA promoters to respond to DNA damage signals. Alternatively, p53 could be actively repressing these lincRNAs in untreated cells followed by a de-repression upon DNA damage.

### Biological properties and sequence features of human p53-regulated lincRNAs

**Figure 4.**
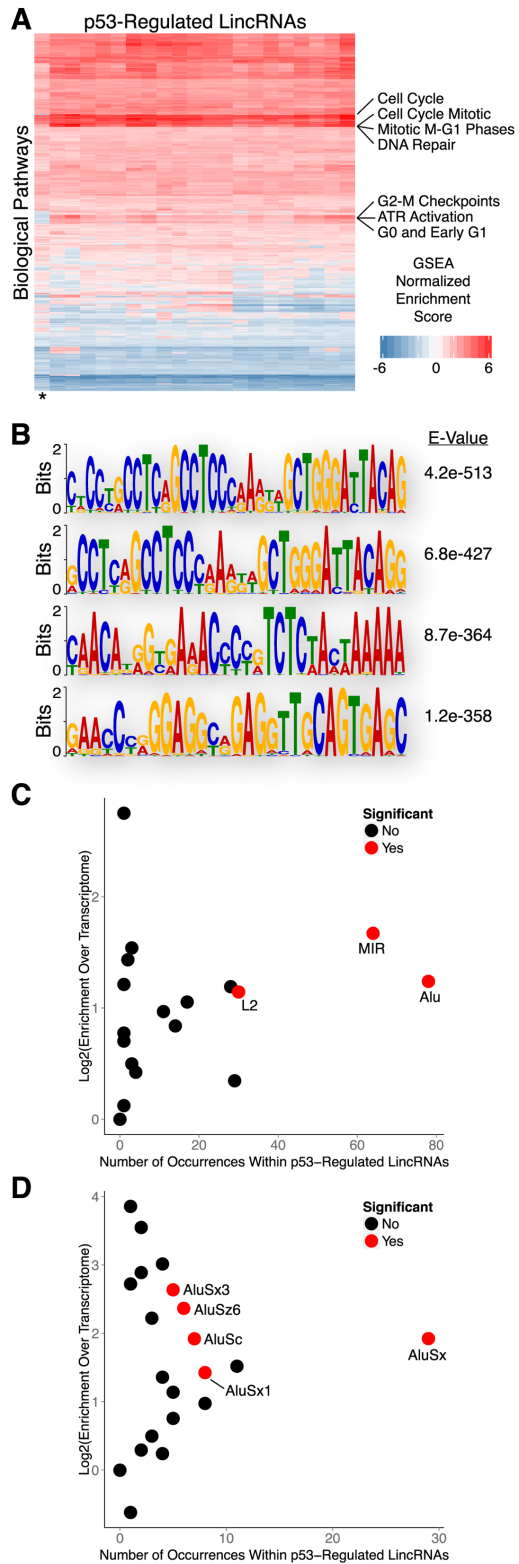
Biological properties and sequence features of human p53-regulated lincRNAs. (**A**) Guilt by association analysis of p53-regulated lincRNAs. (**B**) Sequence motif analysis of lincRNAs regulated by p53. (**C**) Enrichment analysis of transposable element families within lincRNAs regulated by p53. (**D**) Enrichment analysis of Alu family elements within lincRNAs regulated by p53.

To understand the potential biological pathways associated with p53-regulated lincRNAs we performed a ‘guilt by association’ technique that correlates lincRNA and mRNA expression profiles to infer common ontological features. Briefly, we performed gene set enrichment analysis (GSEA) using the gene ontology (GO) terms for protein-coding genes that have expression patterns that are similar to each lincRNA across a compendium of tissue/cell types (21,29). Interestingly, all p53-regulated lincRNAs that were up-regulated in response to DNA damage were strongly associated with biological processes relevant to the DNA damage response, including cell cycle checkpoints and DNA repair (Figure 4A; Supplementary File 2). In contrast, the one p53-regulated lincRNA that was down-regulated (denoted by an * in Figure 4A) had negative associations with many of these processes. While the ‘guilt-by-association’ analysis is a correlation-based approach for inferring lincRNA function as opposed to a direct demonstration of function, our findings provide suggestive evidence that these lincRNAs may be involved in biological pathways relevant to the DNA damage response.

To explore the sequence properties of p53-regulated lincRNAs that may contribute to their biological functions we performed motif analysis using MEME (22,30). We discovered several sequence motifs that were significantly enriched within lincRNA transcripts that were up-regulated in response to DNA damage (Figure 4B). However, none of the enriched motifs resembled consensus binding sequences for known RNA or DNA binding proteins. Intriguingly, all of the identified motifs reside within Alu transposable elements; no motifs were identified when repeat sequences were masked. Subsequent enrichment analysis revealed that p53-regulated lincRNAs are significantly enriched with several families of transposable elements (TEs) relative to the total transcriptome (Figure 4C). Collectively, TEs account for nearly 47% of the total nucleotide coverage of p53-regulated lincRNAs with Alu family elements being the most commonly represented. Further analysis of individual Alu family elements revealed AluSx as the most frequently occurring element within p53-regulated lincRNAs, however other Alu family members were also significantly enriched (Figure 4D). Comparison of p53-regulated lincRNAs to the reference lincRNA catalog, as opposed to the total transcriptome, yielded nearly identical results (Supplementary Figure S2A).

### p53 regulates expression of many lincRNAs in response to DNA damage in primary mouse embryonic fibroblasts

**Figure 5.**
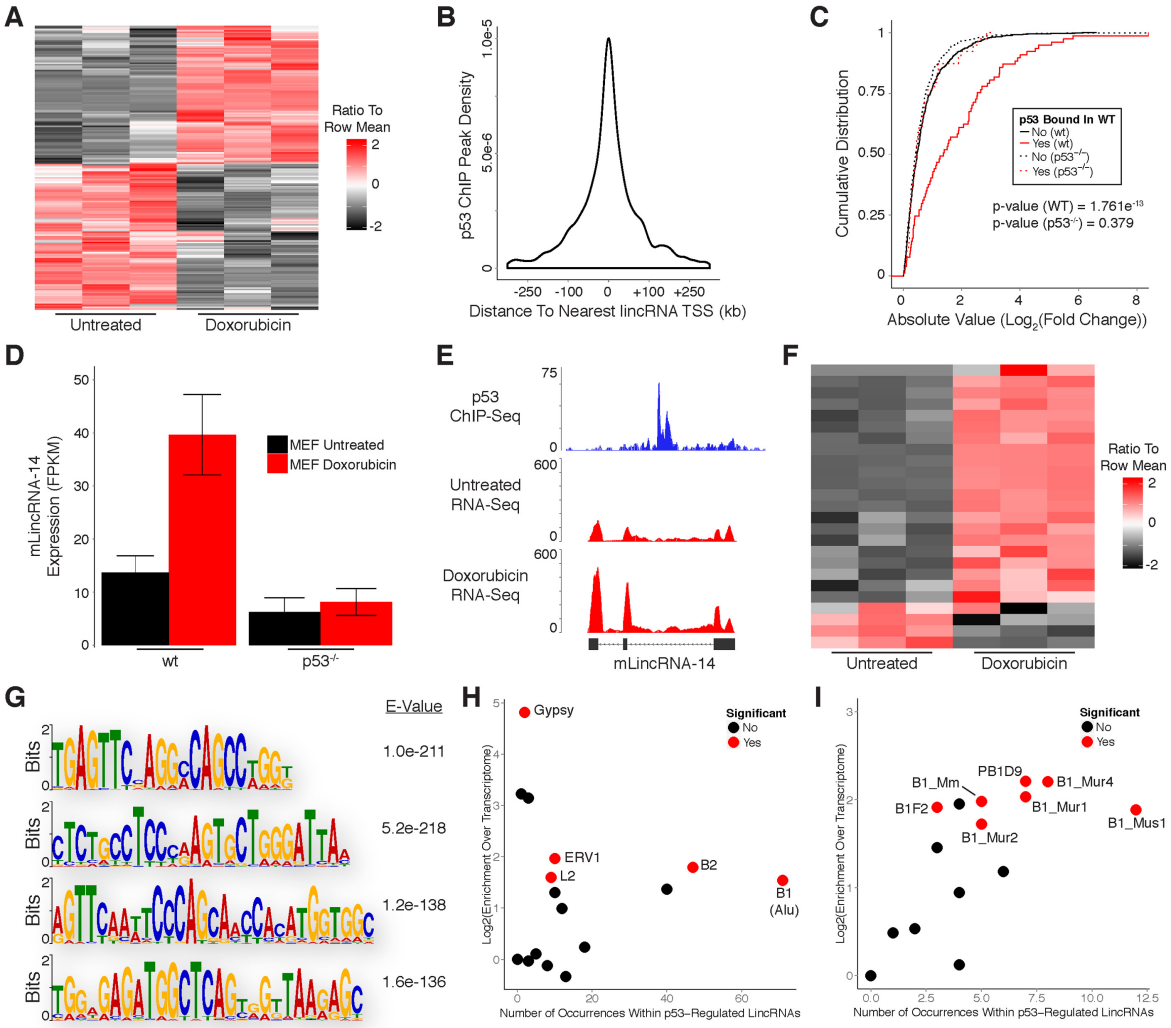
p53 regulates expression of many lincRNAs in response to DNA damage in primary mouse embryonic fibroblasts. (**A**) Heatmap showing differential expression of lincRNAs in response to DNA damage. (**B**) Distribution of p53 ChIP peaks relative to transcription start sites of lincRNAs. (**C**) Cumulative distribution of expression changes for all p53 bound and unbound lincRNAs in response to DNA damage in wild-type and p53^−/−^ primary MEFs. (**D**) RNA-Seq analysis of a p53 target lincRNA in response to DNA damage in wild-type and p53^−/−^ primary MEFs. (**E**) RNA-Seq and p53 ChIP-Seq at selected lincRNA locus. (**F**) Heatmap showing differential expression of direct p53 target lincRNAs in response to DNA damage. (**G**) Sequence motif analysis of lincRNAs regulated by p53. (**H**) Enrichment analysis of transposable element families within lincRNAs regulated by p53. (**I**) Enrichment analysis of B1 family elements within lincRNAs regulated by p53. *P*-values (**5C**) were calculated using the Kolmogorov-Smirnov test.

To investigate the similarities in lincRNA regulation between human and mouse we aligned the RNA-Seq data generated from mouse fibroblasts to a lincRNA reference catalog constructed using our previously described pipeline with publically available RNA-Seq data sets (16). Doxorubicin treatment resulted in up-regulation of 39 lincRNAs and down-regulation of 20 lincRNAs (Figure 5A; Supplementary Table S1). We then used p53 ChIP-Seq to differentiate direct target lincRNAs from those that are indirectly affected by DNA damage. Similar to our observations in human cells, p53 binding sites in MEFs had a wider distribution with respect to the TSS of their nearest respective lincRNA (Figure 5B). However, to remain consistent with our previously analysis of protein-coding gene regulation in MEFs we intersected p53 ChIP peaks with genomic regions spanning from 20 kb upstream to 20 kb downstream of TSSs for annotated lincRNAs. LincRNAs with p53 binding sites within these regions had significantly higher levels of induction/repression in response to DNA damage as compared to lincRNAs without binding sites (Figure 5C). In addition, lincRNAs bound by p53 (in wild-type MEFs) were not induced/repressed in p53^−/−^ MEFs, indicating that they are direct p53 targets (Figure 5C and D). Importantly, the p53-regulated lincRNAs we identified in our RNA-Seq results closely resemble the transcript structures of the lincRNA annotations within our reference catalog (Figure 5E). Relative to human cells, a similar fraction of differentially expressed lincRNAs (21 up-regulated and 4 down-regulated) was directly regulated by p53 in MEFs (Figure 5F; Supplementary Table S1; Supplementary File 1). A subset of the p53 binding sites associated with lincRNA regulation (8/25) were also associated with the regulation of transcripts characterized in our analysis of protein-coding genes in MEFs (Supplementary File 1).

We next searched for sequence motifs that are enriched within the subset of p53-regulated lincRNA transcripts that were up-regulated in response to DNA damage in MEFs. Using MEME we identified several significantly enriched motifs (Figure 5G). There were no obvious similarities between motifs found in human and mouse at the nucleotide level, but many of the motifs identified in mouse lincRNAs occur within B1 TEs which share ancestral history with human Alu elements (31). Enrichment analysis revealed that B1 elements are the most prevalent TE family found within p53-regulated lincRNAs and are significantly enriched relative to the total transcriptome (Figure 5H). The most frequently occurring member of the B1 family was Mus1, although several additional B1 family members were also significantly enriched (Figure 5I). Comparison of p53-regulated lincRNAs to the reference lincRNA catalog, as opposed to the total transcriptome, yielded highly similar results (Supplementary Figure S2B).

### p53 binding occurs predominantly within enhancer regions in primary human fibroblasts

Following our analysis of p53-regulated mRNAs and lincRNAs we found that the majority of p53 binding sites were not associated with direct transcriptional regulation. Only 12.9% (340/2638) of p53 binding sites in human fibroblasts were associated with regulation of nearby transcripts despite our use of large regulatory windows spanning from 30 kb upstream to 30 kb downstream of TSSs. These observations suggest that p53 may have alternative functions within the noncoding genome. The recent development of chromatin state maps has been instrumental in deciphering the regulatory characteristics of genomic regions (32,33). Briefly, chromatin state maps utilize combinatorial patterns of histone modifications to infer the regulatory capacity of genomic elements. We reasoned that these maps could be used to determine the functional importance of p53 binding sites.

To generate a chromatin state map for our analysis we intersected state maps from two independent foreskin fibroblast samples. In addition, we utilized maps from cell types that are functionally distinct from fibroblasts to identify states that are common to a variety of cell types and further increase the stringency of our approach. Because the human fibroblast cell lines in our study were both isolated from fetal skin tissue, we chose maps from keratinocyte lines including normal human epidermal keratinocytes (NHEKs) and two independent foreskin keratinocyte samples. The hybrid chromatin state map used in our study was restricted to genomic regions that have the same predicted chromatin state across all of the aforementioned samples.

**Figure 6.**
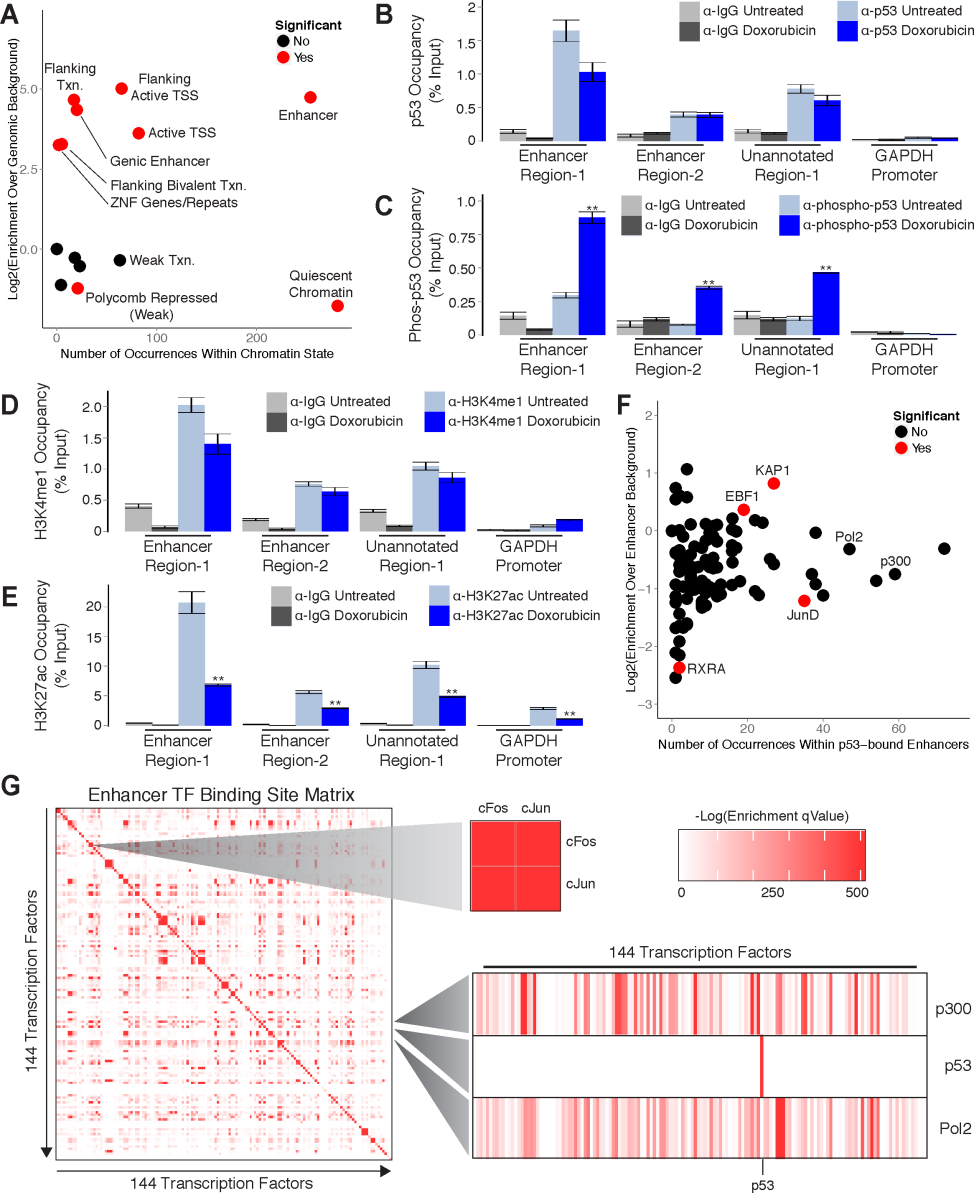
p53 regulation occurs predominantly within enhancer regions in primary human fibroblasts. (**A**) Enrichment analysis of p53 binding sites within annotated chromatin states. (**B**-**E**) ChIP-qPCR analysis of (**B**) p53, (**C**) phospho-p53, (**D**) H3K4me1 and (**E**) H3K27ac at enhancers in response to DNA damage. (**F**) Enrichment analysis of transcription factor binding sites within p53-bound enhancers. (**G**) Global analysis of transcription factor binding site co-occurrence significance within annotated enhancers. Error bars (**6B**, **6C**, **6D** and **6E**) indicate SEM (*n* = 3). *P*-values (**6B**, **6C**, **6D** and **6E**) were calculated using the two-tailed unpaired Student's *t*-test with equal variances. **P < 0.01.

We intersected our experimentally determined p53 binding sites identified using ChIP-Seq with the hybrid chromatin state map and made two notable observations. First, we found that enhancers are among the most common regulatory element in which p53 binding sites occur (Figure 6A; Supplementary File 3). Binding sites are much less frequent in regions of weak transcription, a state that covers 10 times more genomic space than enhancers in our map annotations (Figure 6A). These findings demonstrate that our observations are not a function of the genomic space covered by individual states. Second, p53 binding sites are significantly enriched within enhancer elements when compared to genomic background (Figure 6A). Collectively, these observations indicate that recognition of enhancer elements is a predominant feature of p53 in response to DNA damage.

To validate the role of p53 in enhancer regulation we selected three genomic regions to interrogate using ChIP-qPCR. We termed these regions ‘Enhancer Region-1’ (chr17:67,603,115–67,604,105), ‘Enhancer Region-2’ (chr6:37,212,381–37,213,509) and ‘Unannotated Region-1’ (chr9:84,635,978–84,636,899). Of these regions, ‘Enhancer Region-1’ and ‘Enhancer Region-2’ were annotated as enhancers in our chromatin state map. In contrast, ‘Unannotated Region-1’ was not represented in our state map and was selected to explore the potential functions of p53 binding sites outside of our annotated chromatin states. We first quantified p53 occupancy within these regions in GM00011 fibroblasts cultured in the presence or absence doxorubicin. We were able to detect p53 binding at all three regions in both untreated and doxorubicin treated fibroblasts (Figure 6B). These results suggest that, similar to lincRNA promoters, p53 is actively poised at these sites to respond to DNA damage signals. To test this hypothesis we performed ChIP-qPCR with antibodies against an activated form of p53 that has been phosphorylated at the N-terminal serine-15 (34,35). In response to doxorubicin treatment we observed significant enrichment of activated p53 at all three sites (Figure 6C). These findings confirm that p53 is poised for activation at the examined regions and further indicate that the p53 binding sites identified in this study are actively responding to DNA damage signals.

To verify that the regions we selected for investigation are actually enhancers we next evaluated their local chromatin environment. More specifically, we performed ChIP-qPCR with antibodies that recognize mono-methylated histone-3-lysine-4 (H3K4me1), a hallmark chromatin signature of enhancer activity (36). We detected the presence of H3K4me1 at all three regions in both untreated and doxorubicin treated fibroblasts (Figure 6D). These results confirm that both ‘Enhancer Region-1’ and ‘Enhancer Region-2’ are correctly annotated as enhancers in our chromatin state map. Furthermore, they identify ‘Unannotated Region-1’ as an enhancer and indicate that many of the p53 binding sites that do not overlap regions annotated in our state map may also function at enhancers. To determine if ‘Enhancer Region-1’, ‘Enhancer Region-2’ and ‘Unannotated Region-1’ are functional enhancers in GM00011 fibroblasts we quantified the levels of histone-3-lysine-27 acetylation, a chromatin signature of active enhancers, present at these regions (37). We found that all three regions were marked by H3K27 acetylation in both untreated and doxorubicin treated fibroblasts (Figure 6E). Interestingly, we observed a significant decrease in H3K27 acetylation in response to doxorubicin treatment (Figure 6E). Collectively, these results indicate that p53 binds to functional enhancers and may modulate their regulatory activity.

Enhancers are regulatory elements that influence gene transcription from distant genomic locations (38–41). One hallmark of enhancers is the presence of multiple transcription factor (TF) binding sites that provide a platform for combinatorial gene regulation by multiple factors (42–44). To determine if p53 functions in concert with additional TFs we evaluated TF occupancy within p53-bound enhancers. Using publically available ChIP-Seq data sets collected from a variety of cell types we found that binding sites for several TFs are enriched within p53-bound enhancers relative to all annotated enhancers (Figure 6F). Included within the list of significantly enriched factors is the transcriptional repressor KAP1 which has been previously shown to interact with p53 (45–47). RNA Polymerase II and p300 are among the most frequently occurring factors within p53-bound enhancers, but their occupancy is a general property of enhancer elements explaining their lack of enrichment. Other factors, such as JunD which has been shown to function in opposition to p53, are significantly depleted from p53-bound enhancers (48).

To determine how the enrichment of TFs within p53-bound enhancers compares with global TF co-occupancy we assessed the significance of transcription factor binding site co-occurrence between all factors within all annotated enhancer elements. Intriguingly, p53 is among the few TFs that display no significant co-occurrence with any other factors (Figure 6G). In contrast, binding site co-occurrence of the well-characterized interacting factors cFos and cJun is highly significant. These results suggest that, while specific TFs are significantly enriched within p53-bound enhancers, the co-occurrence of binding sites for these factors is not a distinguishing characteristic of enhancers bound by p53. This observation implies that recognition of enhancers by p53 is not strongly influenced by or dependent on additional TFs and that p53 may have the capacity to act alone as a dominant regulator of enhancer activity.

### p53 binding occurs predominantly within enhancer regions in primary mouse embryonic fibroblasts

The significant enrichment of p53 binding sites within enhancer elements in human fibroblasts prompted us to investigate the relationship between p53 and enhancers in the DNA damage response in mouse fibroblasts. Chromatin state maps have not yet been generated for the mouse genome, however several ChIP-Seq data sets are available that can serve as viable alternatives for inferring chromatin states. We selected ChIP-Seq data generated by the Ludwig Institute for Cancer Research (LICR) as they provided the most comprehensive list of histone and TF ChIP-Seq profiles relevant to our study (49). Included in this list were histone-3-lysine-27-acetlyation (H3K27ac; indicating general regulatory elements), histone-3-lysine-4-monomethylation (H3K4me1; indicating enhancer elements), histone-3-lysine-4-trimethylation (H3K4me3; indicating active promoters), histone-3-lysine-9-acetylation (H3K9ac; indicating active promoters), histone-3-lysine-27-trimethylation (H3K27me3; indicating repressed chromatin) and histone-3-lysine-36-trimethylation (H3K36me3; indicating gene bodies) for heart tissue and embryonic stem cells. In addition, ChIP-Seq profiles for RNA Polymerase II and p300 were available for these cell types. We included histone ChIP-Seq profiles generated from MEFs in our analyses, but corresponding data for p300 were not available in these cells.

**Figure 7.**
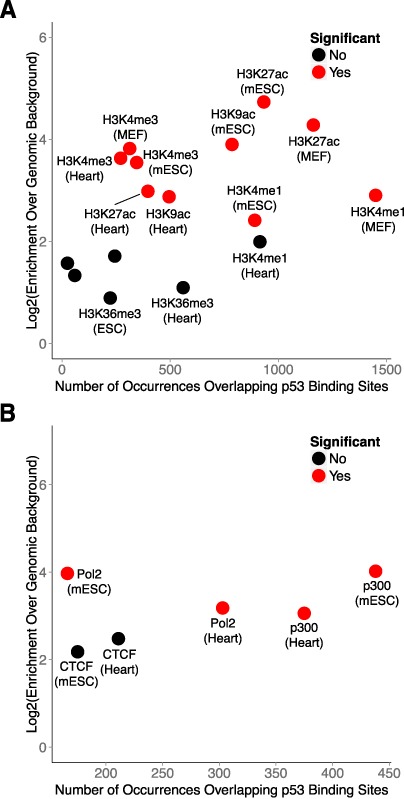
p53 regulation occurs predominantly within enhancer-like regions in primary mouse embryonic fibroblasts. (**A**) Enrichment analysis of p53 binding sites within modified chromatin across several mouse cell types. (**B)** Enrichment analysis of transcription factor co-occurrence with p53 binding sites across several mouse cell types.

We intersected our experimentally determined p53 binding sites from MEFs with the LICR histone ChIP-Seq profiles and found that, similar to human cells, p53 binding occurs most frequently within enhancer (H3K4me1) regions (Figure 7A). Furthermore, enrichment of binding sites within enhancers is comparable to active promoters (H3K4me3 and H3K9ac). Binding sites are most highly enriched within general regulatory regions (H3K27ac), which are inclusive of both active promoters and enhancers. Conversely, enrichment of binding sites within regions of repressed chromatin (H3K27me3) and gene bodies (H3K36me3) was not significant. Collectively, these findings suggest that the prevalence of enhancer recognition by p53 is conserved across mammals.

We next intersected p53 binding sites with the LICR TF ChIP-Seq profiles and observed that binding occurs most frequently in regions associated with p300, a hallmark enhancer-binding protein (Figure 7B). Moreover, the enrichment of binding sites within p300-associated regions is equivalent to RNA Polymerase II-associated regions when compared to genomic background. We observed no significant enrichment of p53 binding sites within CTCF-associated regions, which typically demarcate insulator elements. These findings demonstrate the selectivity of p53 for enhancers as compared to other regulatory elements.

## DISCUSSION

Direct transcriptional regulation of protein-coding genes by p53 in response to DNA damage has been studied for more than 20 years. The application of genome-scale approaches has been particularly useful in elucidating novel aspects of global gene regulation within the p53 network. However, improved genomic technologies/methodologies as well as advances in our understanding of genome regulation provide an opportunity to perform an updated assessment of p53 functions throughout the genome. Here, we have utilized a systematic approach that integrates transcriptome-wide differential expression analysis with genome-wide p53 binding profiles in orthologous human and mouse fibroblast models and have identified many previously unappreciated transcriptional targets of p53. Moreover, we have incorporated the use of chromatin state maps along with additional transcription factor binding profiles and characterized the regulatory properties of p53 binding sites within noncoding regions of the genome.

Alongside canonical p53 targets, our analysis identified a large number of protein-coding genes that were not previously characterized as direct targets of p53 (24,26,50–53). Importantly, our study was performed in primary fibroblasts and the p53 target genes we have identified may represent constituents of the DNA damage response that become improperly regulated in cancer. The majority of these genes displayed relatively low expression, which might also explain why they have eluded detection in previous studies that utilized less sensitive methods. Despite their low expression, regulation by p53 for many of these underappreciated targets is conserved between human and mouse suggesting that they may be important components of the p53 response to DNA damage. Future experimental validation will be necessary to characterize the functional roles of these genes in the DNA damage response.

In addition to protein-coding genes, our analysis identified several lincRNAs that are direct transcriptional targets of p53 in both mouse and human. In contrast to protein-coding genes, we did not find detectable orthology between p53-regulated lincRNAs in human and mouse with respect to either sequence composition or shared synteny. However, there were commonalities in the sequence properties of p53-regulated lincRNAs between species. Specifically, p53-regulated lincRNAs are significantly enriched with Alu TEs in human and the related B1 TEs in mouse. Enrichment of Alu TEs is a distinguishing feature of p53-regulated lincRNAs as previous studies have described a depletion of these elements within lincRNAs in general (54). Alu TEs have been implicated in gene regulation through various mechanisms. For example, Alu elements are enriched within the promoter regions of genes involved in cell proliferation and contain binding sites for multiple TFs that regulate gene expression (55,56). LincRNAs containing Alu sequences could plausibly sequester Alu-binding TFs and inhibit cell proliferation in response to DNA damage (57). Alternatively, noncoding RNAs containing Alu sequences can hybridize to complementary Alu sequences within 3′-untranslated regions (3′ UTRs) of protein-coding transcripts and recruit proteins that induce mRNA decay (58). Alu elements are enriched in 3′ UTRs of transcripts that encode proteins involved in metabolism and signal transduction, making these mRNAs relevant targets for degradation in response to DNA damage (59). Detailed investigation of individual lincRNAs will be required to test these possibilities.

Perhaps the most pronounced observation in our study was the prevalence of p53 binding sites within regulatory enhancer elements. Although recent experimental reports have demonstrated that p53 has the capacity to regulate gene expression through recognition of enhancer elements, the widespread nature of p53 binding to enhancers has not been previously characterized (13,14,60,61). Here, we provide strong evidence that enhancer recognition is actually a predominant feature of the p53 response to DNA damage. The generally accepted capacity of p53 to influence gene expression over large genomic distances is a hallmark attribute of enhancers and strongly supports our observations.

Recognition of enhancer elements by p53 could result in diverse functional outcomes. Two recent reports have characterized individual p53-bound enhancers that interact with distal genes via chromosomal looping and induce gene expression (13,14). In contrast, a study in mouse embryonic stem cells revealed that p53 inhibits the expression of core transcription factors by binding and interfering with the activity of distal enhancers (60). The ability of p53 to inhibit enhancer activity is especially intriguing given our observation that p53-bound enhancers are significantly enriched with KAP1 interacting sites. KAP1 is a transcriptional repressor that interacts with chromatin modifiers and orchestrates heterochromatin formation (62,63). KAP1 binding is typically associated with H3K9-trimethylation, a repressive mark that has been shown to regulate enhancer activity (64). Interestingly, KAP1 lacks a clear DNA binding domain and requires additional factors for recruitment to its targets. Interactions between p53 and KAP1 have been reported, supporting the hypothesis that KAP1 may be an important factor in enhancer repression by p53 (45–47). Although KAP1 is expressed in the fibroblast lines used in this study, we were unable to identify ChIP-grade antibodies despite repeated attempts. Nevertheless, our observation of decreased H3K27 acetylation at p53-bound enhancers in doxorubicin treated fibroblasts is consistent with a potential role for p53 in enhancer repression.

In conclusion, our findings have provided new insight into p53 biology and expanded our understanding of genome regulation by p53. In addition to its roles in the DNA damage response, p53 is an integral component of normal cellular differentiation and development (65). Enhancer regulation by p53 is likely a key factor in these processes as well, a hypothesis that is supported by our finding that p53 binding is significantly enriched within enhancer regions in mouse embryonic stem cells (Figure 7A and B). Furthermore, the biological necessity of p53 has been experimentally validated in organisms as primitive as the sea anemone and it will be interesting to evaluate the prevalence of enhancer regulation by p53 throughout evolution (66).

## ACCESSION NUMBERS

The Gene Expression Omnibus accession number for the RNA-Seq and ChIP-Seq data reported in this paper is GSE55727.

## SUPPLEMENTARY DATA

Supplementary Data are available at NAR Online.

SUPPLEMENTARY DATA
