# Supplementary material for: Integrative genomic analysis reveals widespread enhancer regulation by p53 in response to DNA damage
Source: Nucleic Acids Res. 2015 Apr 16;43(9):4447–62. doi: 10.1093/nar/gkv284 (PMC4482066; doi:10.1093/nar/gkv284)
Supplement: SUPPLEMENTARY DATA [file supp_43_9_4447__index.html]

Integrative genomic analysis reveals widespread enhancer regulation by p53 in response to DNA damage — SUPPLEMENTARY DATA 

# Integrative genomic analysis reveals widespread enhancer regulation by p53 in response to DNA damage

## SUPPLEMENTARY DATA

**Files in this Data Supplement:**

- SUPPLEMENTARY DATA
- SUPPLEMENTARY DATA
- SUPPLEMENTARY DATA
- SUPPLEMENTARY DATA
