# Supplementary material for: Integrative genomic analysis reveals widespread enhancer regulation by p53 in response to DNA damage
Source: Nucleic Acids Res. 2015 Apr 16;43(9):4447–62. doi: 10.1093/nar/gkv284 (PMC4482066; doi:10.1093/nar/gkv284)
Supplement: SUPPLEMENTARY DATA [file supp_gkv284_nar-03119-d-2014-File012.pdf]

Integrative Genomic Analysis Reveals Widespread Enhancer Regulation by p53 in Response to DNA Damage

Scott T. Younger[1,2], Daniela Kenzelmann-Broz[3], Heiyoun Jung[3], Laura D. Attardi[3], and John L. Rinn[1,2,*]

[1]Department of Stem Cell and Regenerative Biology, Harvard University, Cambridge, MA 02138, USA. [2]Broad Institute of MIT and Harvard, Cambridge, MA 02142, USA. [3]Division of Radiation and Cancer Biology, Department of Radiation Oncology, Stanford University School of Medicine, Stanford, CA USA.

Supplementary Information

Supplementary Table 1. Summary of differential gene expression in human and mouse primary fibroblasts in response to doxorubicin treatment.

Supplementary Table 2. PCR Primers and PCR conditions.

Supplementary File 1. Summary of the p53-regulated transcriptome: gene names, genomic coordinates, expression values, fold changes, and corresponding p53 binding sites for p53-regulated transcripts (mRNAs and lincRNAs) in human and mouse primary fibroblasts in response to doxorubicin treatment.

Supplementary File 2. Summary of guilt-by-association predictions for p53-regulated lincRNA functions: gene set enrichment analysis (GSEA) output.

Supplementary File 3. Summary of p53 binding sites within annotated chromatin states: coordinates of p53 binding sites and corresponding chromatin state annotations.

Supplementary Figure 1. Comparison of p53 binding sites across multiple cell types and treatments. (**A-C**) Comparison of p53 binding sites characterized in this study with sites identified in previous studies using various forms of p53 activation in (**A**) MCF7, (**B**) lymphoblastoid, and (**C**) U2OS cells. (**D**) Comparison of p53 binding sites in doxorubicin treated human fetal fibroblasts (this study), lymphoblastoid cells, and U2OS cells.

Supplementary Figure 2. Enrichment analysis of Alu/B1 family elements within lincRNAs regulated by p53 relative to reference lincRNA catalogs in (**A**) primary human fibroblasts (Alu) and (**B**) primary mouse embryonic fibroblasts (B1).

Supplementary Figure 1

A

Younger et al.

Nikulenkov et al. Cell Death Differ. 2012 Dec;19(12):1992-2002.

Doxorubicin (hFetal Fibro.) : 2,638
Nutlin (MCF7) : 17,958
Overlap : 1,652

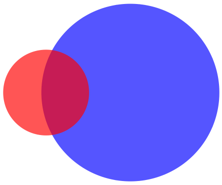

Doxorubicin (hFetal Fibro.) : 2,638
5FU (MCF7) : 11,320
Overlap : 1,366

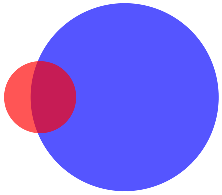

Doxorubicin (hFetal Fibro.) : 2,638
RITA (MCF7) : 11,477
Overlap : 1,707

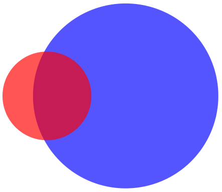

B

Younger et al.

Zeron-Medina et al. Cell. 2013 Oct 10;155(2):410-22.

Doxorubicin (hFetal Fibro.) : 2,638
Nutlin (Lymphoblastoid) : 1,183
Overlap : 351

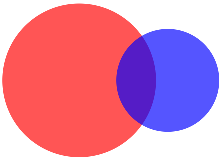

Doxorubicin (hFetal Fibro.) : 2,638
Doxorubicin (Lymphoblastoid) : 13,055
Overlap : 1,668

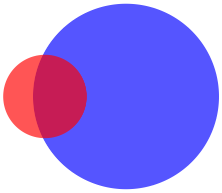

Doxorubicin (hFetal Fibro.) : 2,638
IR (Lymphoblastoid) : 237
Overlap : 161

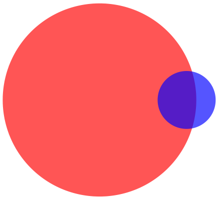

C

Younger et al.

Menendez et al. Nucleic Acids Res. 2013 Aug;41(15):7286-301.

Doxorubicin (hFetal Fibro.) : 2,638
Nutlin (U2OS) : 18,156
Overlap : 2,226

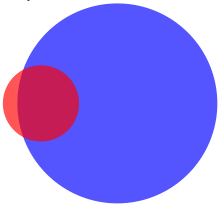

Doxorubicin (hFetal Fibro.) : 2,638
Doxorubicin (U2OS) : 3,087
Overlap : 1,555

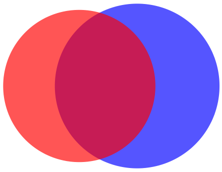

D

All Doxorubicin Samples (Diagram Not Scaled)

Doxorubicin (hFetal Fibro.) : 2,638
Doxorubicin (Lymphoblastoid) : 13,055
Doxorubicin (U2OS) : 3,087

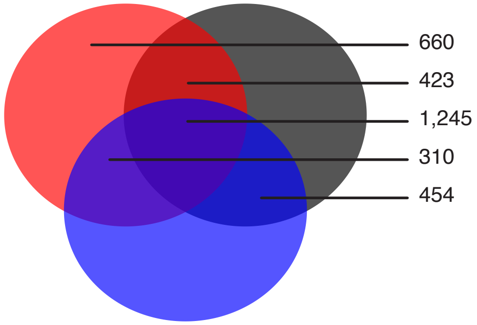

Supplementary Figure 2

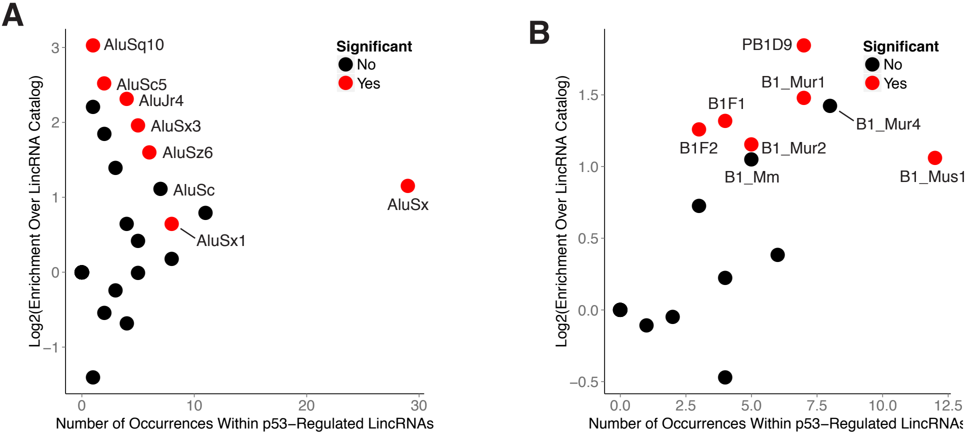

Supplementary Table 1. Summary of differential gene expression in response to doxorubicin treatment.

		Human		Mouse	
		**mRNA**	**lincRNA**	**mRNA**	**lincRNA**
**Up-regulated**	**Total**	**1365**	**101**	**3124**	**94**
	**p53 Targets**	**262 (19%)**	**22 (22%)**	**585 (19%)**	**21 (22%)**
**Down-regulated**	**Total**	**1598**	**32**	**3334**	**99**
	**p53 Targets**	**106 (6.6%)**	**1 (3.1%)**	**175 (5.2%)**	**4 (4%)**

Supplementary Table 2. PCR Primers and PCR conditions.

Target	Direction	Use	Sequence
LincRNA-7	Forward	RT-qPCR	TGCTGCATTTACAGGTGAGG
LincRNA-7	Reverse	RT-qPCR	AGGATGGCAGGTACATGAGG
LincRNA-4	Forward	RT-qPCR	GGAGCCTAGCATCACTCGTC
LincRNA-4	Reverse	RT-qPCR	CTCCTCTTCCAGCAGTTTGG
LincRNA-1	Forward	RT-qPCR	AAGCCAAGCTTCCTGTTTCA
LincRNA-1	Reverse	RT-qPCR	GGGTCTAGGGGATGGGAATA
LincRNA-7 Promoter	Forward	ChIP-qPCR	GCACGGACTGAGAAACACAA
LincRNA-7 Promoter	Reverse	ChIP-qPCR	GGGGCATTTGGAGATTTTG
LincRNA-4 Promoter	Forward	ChIP-qPCR	ACTTACCATGGGCAGTCAGC
LincRNA-4 Promoter	Reverse	ChIP-qPCR	GGGTGGTGGGTGTCACTAAA
LincRNA-1 Promoter	Forward	ChIP-qPCR	AGTGGACTGGAGCCTGACAT
LincRNA-1 Promoter	Reverse	ChIP-qPCR	AGCACCACCTCACACATTGA
Enhancer Region-1	Forward	ChIP-qPCR	CCACAGGCTGCACAAGATAA
Enhancer Region-1	Reverse	ChIP-qPCR	TCTGAAAGTGATGCTGTGGAA
Enhancer Region-2	Forward	ChIP-qPCR	GCCAGGCAATTTTGTTGATT
Enhancer Region-2	Reverse	ChIP-qPCR	TAGCCTGCTGGATGATGAAA
Unannotated Region-1	Forward	ChIP-qPCR	AGGCAGTGGAGAGTGGAGAA
Unannotated Region-1	Reverse	ChIP-qPCR	GACCCTTGCAACCTGCTTAG
GAPDH Promoter	Forward	ChIP-qPCR	TACTAGCGGTTTTACGGGCG
GAPDH Promoter	Reverse	ChIP-qPCR	TCGAACAGGAGGAGCAGAGAGCGA

PCR Conditions	Denature	Annealing	Extension	Cycles
RT-qPCR	95°C, 20 s	57°C, 30 s	72°C, 45 s	x40
ChIP-qPCR	95°C, 20 s	57°C, 30 s	72°C, 45 s	x40
